# Discordance between patient and physician assessments of global disease activity in rheumatoid arthritis and association with work productivity

**DOI:** 10.1186/s13075-016-1004-3

**Published:** 2016-05-21

**Authors:** Josef S. Smolen, Vibeke Strand, Andrew S. Koenig, Annette Szumski, Sameer Kotak, Thomas V. Jones

**Affiliations:** Division of Rheumatology, Medical University of Vienna and Hietzing Hospital, Vienna, Austria; Division of Immunology/Rheumatology, Stanford University School of Medicine, Palo Alto, CA USA; Pfizer, Collegeville, PA USA; inVentiv Health, Princeton, NJ USA; Pfizer, New York, NY USA

**Keywords:** PRESERVE, Discordance, Rheumatoid arthritis, Work productivity, Etanercept

## Abstract

**Background:**

Discordance between patient and physician ratings of rheumatoid arthritis (RA) severity occurs in clinical practice and correlates with pain scores and measurements of joint disease. However, information is lacking on whether discordance impacts patients’ ability to work. We evaluated the discordance between patient and physician ratings of RA disease activity before and after treatment with etanercept and investigated the associations between discordance, clinical outcomes, and work productivity.

**Methods:**

In the PRESERVE clinical trial, patients with moderate RA received open-label etanercept 50 mg once weekly plus methotrexate for 36 weeks. Baseline and week-36 disease characteristics and clinical and work productivity outcomes were categorized according to week-36 concordance category, defined as positive discordance (patient global assessment – physician global assessment ≥2), negative discordance (patient global assessment – physician global assessment ≤ –2), and concordance (absolute difference between the two disease activity assessments = 0 or 1). Correlations between discordance, clinical outcomes, and predictors of discordance were determined.

**Results:**

At baseline, 520/762 (68.2 %) patient and physician global assessment scores were concordant, 194 (25.5 %) were positively discordant, and 48 (6.3 %) were negatively discordant. After 36 weeks of therapy, 556/763 (72.9 %) scores were concordant, 189 (24.8 %) were positively discordant, and 18 (2.4 %) were negatively discordant. Patients with week-36 concordance had the best 36-week clinical and patient-reported outcomes, and overall, the greatest improvement between baseline and week 36. Baseline pain, swollen joint count, duration of morning stiffness, fatigue, and patient general health significantly correlated with week-36 discordance, *p* < 0.0001 to *p* < 0.05. Additionally, baseline pain, patient general health, and C-reactive protein were predictors of week-36 discordance (odds ratios 1.22, 1.02, and 0.98, respectively). For the employed patients, percent impairment while working and percent overall work impairment were highest (greatest impairment) at baseline and 36 weeks in the group with positive discordance.

**Conclusions:**

The percentage of patients with concordance increased after 36 weeks of treatment with etanercept, with concordant patients demonstrating the greatest improvement in clinical and patient-reported outcomes. Discordance correlated with several measures of disease activity and was associated with decreased work productivity.

**Trial registration:**

ClinicalTrials.gov identifier: NCT00565409. Registered 28/11/2007

## Background

Assessments of disease activity may differ between patients and physicians. Studies have shown that a discrepancy occurs in approximately one third of cases; however, this can vary depending on how it is measured [1–6]. Discordance occurs in many chronic illnesses, including rheumatoid arthritis (RA), and may be present before treatment starts or during the course of therapy [1–15]. In RA, physicians tend to rate disease activity lower than patients; however, scoring differences in both directions have been reported [1–7, 9, 11–15]. Several reasons have been proposed for these differences, including functional impairment, limited knowledge about health, and differences in the signs and symptoms on which patients and physicians focus. Importantly, patients primarily concentrate on pain and tenderness when rating RA disease activity, and physicians focus on swollen joints and C-reactive protein (CRP) or erythrocyte sedimentation rate (ESR) [3–5, 9, 11–13, 15, 16]. As pain may be present even if the disease is controlled, the difference between patient and physician viewpoints may be exaggerated when the disease is not active [15]. This may be the case particularly in long-standing disease when joint damage and damage-related disability are present. It is also possible that patients self-assess signs and symptoms of other comorbidities and attribute them to their RA. Finally, physicians may relate the condition of one patient to that of the many patients seen over the years, while individual patients may have little more than their own situation and wellbeing as comparator.

A better understanding of patient and physician bias (conscious and unconscious), perceptions, judgment, beliefs about what medicines can or cannot do, and disconnects in the assessment of RA signs and symptoms may enable clinicians to more effectively communicate with patients, improve treatment outcomes, and help patients achieve their varied treatment goals [16]. This includes a component of active listening and openness to patient input. In other chronic diseases, such strategies, including open-ended discussions with patients, have demonstrated an increased ability to identify patients at risk of non-adherence and allow for better alignment with patient goals [17, 18].

Decrease in pain and improvement of physical function may not be the only objectives for patients. Patients may also be concerned about the impact of RA on their overall activities and their ability to work. Studies have demonstrated that RA, whether early or late in the disease course, affects patients’ ability to work, which in turn can lead to presenteeism, absenteeism, unemployment, income loss, and early retirement [19–29].

To our knowledge, no published studies have evaluated whether discordance in the assessment of disease activity is connected to presenteeism, absenteeism, and work impairment in patients with RA. In addition, differences in assessments of disease activity in RA have not been rigorously examined in clinical trial populations. Publications to date have evaluated mostly clinic patients in observational studies; consequently, the results are often complicated by the fact that the patients did not receive identical therapy or monitoring and came from a single center and/or academic environment, in contrast to a large, multicenter clinical trial [1–7, 9, 11–13, 15].

The PRESERVE trial (ClinicalTrials.gov identifier, NCT00565409) was a multicenter two-period trial in adults with moderate RA [30]. Period 1 was the open-label, single treatment phase of the trial that evaluated responses to combination etanercept and methotrexate therapy for 36 weeks, and period 2 was the randomized, double-blind phase that investigated the outcomes of dose reduction or withdrawal of etanercept. This analysis only includes data from period 1, thereby focusing on discordance in patients with moderate disease activity who were being treated to a target of low disease activity. This report examines (1) the difference, i.e., discordance, between patients’ and physicians’ global assessments of disease activity at baseline and 36 weeks; (2) correlations between clinical parameters and discordance of global assessments at baseline and week 36; (3) baseline predictors of week-36 discordance; and (4) whether week-36 discordance is associated with work productivity.

## Methods

Details of the PRESERVE trial have been presented elsewhere [30]. In brief, patients were 18–70 years of age with a diagnosis of RA based on the 1987 American College of Rheumatology criteria. Patients had a moderate disease activity score based on a 28-joint count (DAS28 >3.2 and ≤5.1) and were enrolled at 80 centers in Europe, Asia, Australia, and Latin America. Eligible patients were required to have taken stable doses of oral methotrexate 15–25 mg/week for at least 8 weeks prior to receiving 50 mg open-label etanercept once weekly plus methotrexate ≥10 mg/week for the 36 weeks of period 1. Exclusion criteria included use of disease-modifying anti-rheumatic drugs (DMARDs) other than methotrexate within 28 days before baseline or current or previous use of a biologic DMARD for RA.

The study was conducted in accordance with the International Conference on Harmonisation guideline for good clinical practice and the ethical principles of the Declaration of Helsinki. Written informed consent was obtained from all patients prior to enrollment. The study protocol and all consent forms were reviewed and approved by an institutional review board or an independent ethics committee at each participating center (see “Acknowledgments” for details).

Patients and physicians completed their respective global assessments of disease activity at baseline and weeks 4, 8, 12, 20, 28, and 36. Patients were blinded to physician global assessments and physicians were blinded to patient global assessments. The physician global assessment was performed prior to the physician having access to the C-reactive protein (CRP) levels from that visit. The global assessments used a numerical rating scale on which respondents were asked to rate disease activity by circling a number ranging from 0 (no disease activity) to 10 (extreme disease activity) [31]. At baseline and week 36, mean differences between patient and physician scores were categorized as positive discordance (patient global assessment – physician global assessment ≥2), negative discordance (patient global assessment – physician global assessment ≤ –2), or concordance (absolute difference between the two disease activity scores = 0 or 1). The cutoff of 2 was chosen by rounding to the closest whole number above the one standard deviation of 1.72 obtained from the mean difference between patient and physician global assessments.

Concordance/discordance was determined for all patients, and for the subgroup of patients who achieved all three outcomes of swollen joint count (SJC) ≤1, tender joint count (TJC) ≤1, and CRP ≤1 mg/dL at 36 weeks, and for those patients who achieved the Boolean-based definition of clinical remission (SJC ≤1, TJC ≤1, CRP ≤1 mg/dL, and patient global assessment ≤1) [32]. Concordance/discordance was also determined for the patients who achieved remission according to the clinical disease activity index (CDAI ≤2.8) at 36 weeks. Additionally, the rates of Boolean and CDAI remission were evaluated according to baseline and week-36 discordance status.

Endpoints at 36 weeks according to concordance/discordance category included DAS28, CRP, TJC, SJC, health assessment questionnaire disability index (HAQ-DI), erythrocyte sedimentation rate (ESR), patient and physician global assessments, duration of morning joint stiffness, brief pain inventory (BPI), simplified disease activity index (SDAI), CDAI, patient general health visual analog scale (VAS), and functional assessment of chronic illness therapy-fatigue (FACIT-fatigue). Additionally, for the subgroup of patients who achieved the three outcomes of SJC ≤1, TJC ≤1, and CRP ≤1 mg/dL at 36 weeks, the measurement of the modified total Sharp score was characterized by concordance category.

Patient global assessment and patient general health VAS are assessments with several important differences. The patient global assessment requests that patients measure overall arthritis activity by asking them to circle a number between 0 and 10, with 0 indicating no arthritis activity and 10 indicating extreme activity. The patient general health VAS asks patients to indicate, “in general how would you rate your health over the last 2–3 weeks?” Patients place a mark on a 100-mm line, with 0 mm meaning “very well” and 100 mm meaning “extremely bad.” Thus, the patient global assessment measures arthritis disease activity and the patient general health VAS measures overall health.

Additionally, the extent to which work productivity activity impairment was associated with discordance was determined for each component of the work productivity activity impairment questionnaire for RA (WPAI:RA). The WPAI:RA is a validated tool for measuring work productivity that was used in the PRESERVE trial [33], consisting of four components: activity impairment, absenteeism, presenteeism, and overall work impairment [34, 35]. The questionnaire focuses on impairment due to RA only. Activity impairment includes patients not employed outside the home; the other components are measured for employed patients only. Absenteeism is missed work days due to health, and presenteeism is a reduction of productivity or diminished work capacity while at work [35]. Overall work impairment is calculated using both absenteeism and presenteeism. Each component of the WPAI:RA is scored from 0–100 %; higher scores indicate a worse outcome.

### Statistical analysis

The analyses included patients in the period 1 population who received at least one dose of study medication and had data at baseline and week 36. Missing data at week 36 were not imputed, rather the observed case approach was used. Descriptive statistics were used to characterize differences in patient demographics, disease characteristics, and clinical endpoints among concordant and discordant groups. *P* values for demographic and baseline disease characteristics were generated using the *F* test from analysis of variance for continuous variables; Cochran-Mantel-Haenszel test or Fisher’s exact test was used for categorical variables. The proportion of patients who shifted between baseline and week-36 concordance categories was determined.

Correlations between discordance (analyzed as continuous parameters) and clinical endpoints were determined using Pearson’s *r* correlation. Stepwise logistic regression was performed to determine significant baseline predictors of week-36 discordance. As binomial logistic regression requires two categories, the concordant and negative discordance groups were combined into one category and compared with positive discordance. The following parameters were included in the stepwise logistic regression analyses of baseline predictors: age, sex, disease duration, race, prior alcohol and tobacco use, rheumatoid factor status, body mass index, TJC-28, SJC-28, CRP, ESR, HAQ-DI, DAS28, CDAI, SDAI, FACIT-fatigue, patient general health, BPI, and duration of morning stiffness. Odds ratios were calculated to describe the strength of the association between baseline or week-36 parameters and the two discordance categories at 36 weeks.

Descriptive statistics for change from baseline were determined for the WPAI:RA for each concordance group. Three of the four outcome scores (absenteeism, presenteeism, and overall work impairment due to RA) were evaluated for the subgroup of patients who were employed at both baseline and 36 weeks; activity impairment was measured for the full population included in this analysis.

## Results

### Patients

The PRESERVE trial enrolled 834 patients [30]. The first period (36 weeks) of the study was completed by 756 patients (90.6 %); 77 patients discontinued and results from one patient were not included due to a data discrepancy. This analysis includes patients in the period-1 population with data to week 36 (n = 763). There were 13 patients who did not complete period 1 but had week-36 data (e.g., their 28-week visit occurred late and was assigned to week 36), and 6 patients completed period 1 but did not have week-36 data (e.g., their week-36 visit occurred early and was assigned to week 28); thus, the difference of 7 patients between period-1 completers and patients with week-36 data. Mean (SD) age was 48.2 (11.9) years, 82.8 % were female, 74.4 % were white, and duration of RA symptoms was 7.0 (6.9) years. Additional baseline disease characteristics are provided in Table 1.

**Table 1:** Demographics and baseline disease characteristics according to week-36 concordance category

	Week 36 (n = 763)		
	Positive discordance (n = 189)	Concordance (n = 556)	Negative discordance (n = 18)
Age, years, mean (SD)	48.8 (11.5)	47.9 (12.1)	49.8 (10.8)
Female, *n* (%)	160 (84.7)	455 (81.8)	17 (94.4)
White, *n* (%)	130 (68.8)	424 (76.3)	14 (77.8)
Prior alcohol, *n* (%)	22 (11.6)	64 (11.5)	2 (11.1)
Prior tobacco, *n* (%)	39 (20.6)	98 (17.6)	4 (22.2)
Rheumatoid factor+, *n* (%)	130 (68.8)	411 (73.9)	11 (61.1)
Duration of disease symptoms, years, mean (SD)	6.8 (6.9)	6.9 (6.9)	9.0 (8.1)
Weekly dose of methotrexate, mg, mean (SD)	16.4 (2.4)	16.5 (2.8)	15.8 (1.9)
Prior treatment, *n* (%)			
Methotrexate	189 (100.0)	556 (100.0)	18 (100.0)
DMARDs other than methotrexate	46 (24.3)	145 (26.1)	8 (44.4)
Glucocorticoids	117 (61.9)	325 (58.5)	10 (55.6)
NSAIDs	133 (70.4)	422 (75.9)	14 (77.8)

Positive discordance: patient global assessment – physician global assessment ≥2. Negative discordance: patient global assessment – physician global assessment ≤ –2. Concordance: patient global assessment – physician global assessment = 0 or 1. *SD* standard deviation, *DMARDs* disease-modifying anti-rheumatic drugs, *NSAIDs* nonsteroidal anti-inflammatory drugs

### Concordance

At baseline, 520/762 patient and physician global assessment scores (68.2 %; one patient had no baseline data available for one of the measures) were concordant (i.e., the difference between the scores was 0 (34.3 %) or 1 (34.0 %)). The number of patients with positively and negatively discordant scores was 194 (25.5 %) and 48 (6.3 %), respectively. Table 2 lists disease characteristics at baseline, week 36, and the change between baseline and week 36 for all patients, according to week-36 concordance category. At baseline, several clinical and patient-reported characteristics differed significantly according to concordance/discordance category: CRP, SJC-28, duration of morning stiffness, BPI, FACIT-fatigue, CDAI, SDAI, patient and physician global assessment, and patient general health. The values for most of these characteristics indicated more severe disease in the discordant than in the concordant patients. The exception was CRP; this was highest in the patients with concordance, with a mean (SD) of 13.4 (17.7) mg/L versus 9.9 (13.9) mg/L for positive discordance and 8.1 (7.1) mg/L for negative discordance.

**Table 2:** Disease characteristics at baseline and week 36, according to week-36 concordance category

	Week 36 (n = 763)				
	Time frame	Positive discordance (n = 189)	Concordance (n = 556)	Negative discordance (n = 18)	*P* value^a^
DAS28	BL	4.4 (0.4)	4.4 (0.4)	4.5 (0.4)	0.2356
	Week 36	2.8 (1.1)	2.2 (0.9)	3.1 (1.3)	<0.0001
	Δ BL to wk 36	−1.6 (1.0)	−2.2 (0.9)	−1.4 (1.3)	<0.0001
CRP, mg/L	BL	9.9 (13.9)	13.4 (17.7)	8.1 (7.1)	0.0256
	Week 36	6.4 (8.2)	6.4 (7.9)	7.6 (12.0)	0.8036
	Δ BL to wk 36	−3.4 (14.7)	−7.0 (17.0)	−0.4 (9.8)	0.0123
CRP, mg/L, median	BL	4.6	5.9	4.0	
	Week 36	4.0	4.0	4.0	
	Δ BL to wk 36	0	−1	0	
ESR, mm/h	BL	21.0 (13.9)	22.9 (13.1)	20.2 (10.5)	0.1652
	Week 36	13.4 (13.1)	11.8 (9.5)	15.0 (10.5)	0.1083
	Δ BL to wk 36	−7.6 (11.2)	−11.1 (12.6)	−5.2 (10.7)	0.0006
TJC (0–28)	BL	5.0 (2.6)	5.0 (2.8)	6.0 (3.5)	0.3502
	Week 36	1.8 (2.5)	1.1 (2.2)	4.0 (5.6)	<0.0001
	Δ BL to wk 36	−3.3 (3.4)	−4.0 (3.2)	−2.0 (6.3)	0.0039
SJC (0–28)	BL	3.5 (2.3)	4.0 (2.7)	4.8 (3.3)	0.0227
	Week 36	1.1 (1.9)	0.9 (1.9)	3.9 (4.3)	<0.0001
	Δ BL to wk 36	−2.3 (2.5)	−3.1 (2.6)	−0.9 (5.0)	<0.0001
Duration of morning joint stiffness, min	BL	227 (384)	158 (314)	242 (472)	0.0453
	Week 36	78 (164)	39 (166)	67 (144)	0.0191
	Δ BL to wk 36	−148 (370)	−120 (324)	−175 (515)	0.5315
BPI (0–10)	BL	4.7 (2.1)	3.7 (1.9)	3.9 (2.1)	<0.0001
	Week 36	3.2 (2.2)	1.1 (1.3)	1.8 (1.6)	<0.0001
	Δ BL to wk 36	−1.5 (2.4)	−2.6 (2.1)	−2.1 (2.5)	<0.0001
HAQ-DI (0–3)	BL	1.2 (0.6)	1.1 (0.6)	1.2 (0.7)	0.1458
	Week 36	0.9 (0.6)	0.5 (0.5)	0.7 (0.6)	<0.0001
	Δ BL to wk 36	−0.3 (0.6)	−0.7 (0.5)	−0.5 (0.5)	<0.0001
FACIT-fatigue^b^ (0–52)	BL	30.8 (9.3)	33.7 (9.4)	29.7 (10.0)	0.0006
	Week 36	35.7 (9.5)	43.1 (8.0)	39.1 (9.8)	<0.0001
	Δ BL to wk 36	4.9 (9.4)	9.4 (8.8)	9.3 (11.4)	<0.0001
CDAI (0–76)	BL	18.1 (4.5)	17.6 (5.0)	21.2 (6.3)	0.0049
	Week 36	8.7 (5.8)	4.7 (5.1)	13.6 (10.1)	<0.0001
	Δ BL to wk 36	−9.5 (6.5)	−12.9 (6.4)	−7.6 (12.0)	<0.0001
SDAI (0–86)	BL	19.2 (4.6)	18.9 (5.2)	22.0 (6.2)	0.0382
	Week 36	9.4 (5.9)	5.3 (5.3)	14.4 (10.5)	<0.0001
	Δ BL to wk 36	−9.8 (6.4)	−13.6 (6.5)	−7.6 (12.2)	<0.0001
Patient global assessment (0–10)	BL	5.5 (1.6)	4.6 (1.7)	5.2 (2.0)	<0.0001
	Week 36	4.5 (1.8)	1.5 (1.2)	1.4 (1.1)	<0.0001
	Δ BL to wk 36	−1.0 (2.2)	−3.1 (1.9)	−3.8 (2.2)	<0.0001
Physician global assessment (0–10)	BL	4.2 (1.4)	4.0 (1.3)	5.2 (1.5)	0.0002
	Week 36	1.4 (1.2)	1.3 (1.1)	4.3 (2.1)	<0.0001
	Δ BL to wk 36	−2.9 (1.6)	−2.7 (1.5)	−0.9 (2.9)	<0.0001
Patient general health VAS	BL	47.8 (16.9)	40.9 (15.9)	45.7 (22.8)	<0.0001
	Week 36	36.9 (19.1)	12.4 (13.3)	14.7 (14.4)	<0.0001
	Δ BL to wk 36	−11.1 (22.4)	−28.5 (18.8)	−31.1 (22.6)	<0.0001

Values are mean (SD). Positive discordance: patient global assessment – physician global assessment ≥2. Negative discordance: patient global assessment – physician global assessment ≤ –2. Concordance: patient global assessment – physician global assessment = 0 or 1. *BL* baseline, *BPI* brief pain inventory, *CDAI* clinical disease activity index, *CRP* C-reactive protein, *DAS28* disease activity score based on 28 joint count, *ESR* erythrocyte sedimentation rate, *FACIT-fatigue* functional assessment of chronic illness therapy-fatigue, *HAQ-DI* health assessment questionnaire disability index, *SDAI* simplified disease activity index, *SJC* swollen joint count, *TJC* tender joint count, *VAS* visual analog scale, *wk*, week. ^a^ *F*-statistic (analysis of variance). ^b^Higher scores indicate less fatigue

After 36 weeks of therapy, concordance increased to 556/763 patients (72.9 %); the number of patients with positively and negatively discordant scores decreased to 189 (24.8 %) and 18 (2.4 %), respectively. Improvement between baseline and week 36 differed significantly according to concordance/discordance status for DAS28, CRP, ESR, TJC, SJC, BPI, HAQ-DI, FACIT-fatigue, CDAI, SDAI, patient and physician global assessment, and patient general health (*p* < 0.05 for change in CRP and TJC; *p* < 0.001 for ESR; and *p* < 0.0001 for all others; Table 2). Patients with concordant scores at week 36 had the best 36-week clinical and patient-reported outcomes, and for most measurements, the greatest improvement between baseline and week 36, compared with patients with positively or negatively discordant scores.

At week 36, patients with negative discordance exhibited the highest values of DAS28, CRP, ESR, TJC, SJC, CDAI, and SDAI, suggesting that physicians, more than their patients, may have looked at “objective” disease activity measures when determining their global assessment. In contrast, patients with positive discordance had the longest morning joint stiffness and worst values for BPI, HAQ-DI, FACIT-fatigue, and patient general health, suggesting that they, more than their physicians, focused on subjective outcomes when determining their global assessment.

**Fig. 1:**
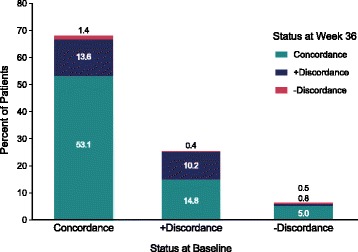
Shifts in discordance categories, baseline to week 36 (n = 762). Positive discordance: patient global assessment – physician global assessment ≥2. Negative discordance: patient global assessment – physician global assessment ≤ –2. Concordance: patient global assessment – physician global assessment = 0 or 1

Most patients who were concordant at the beginning of the study remained concordant at 36 weeks (Fig. 1). For the patients with positive or negative discordance at baseline, the greatest shift at 36 weeks was to concordance. The smallest shift from any category was to negative discordance.

### Disease remission

Subgroup analysis results indicated that 442/755 patients (58.5 %) achieved SJC and TJC ≤1 and CRP ≤1 mg/dL at week 36. Of those 442 patients, 255 (57.7 %) also had a patient global assessment ≤1, thus meeting the Boolean-based criteria for clinical remission. Interestingly, the remaining 187 patients (42.3 %) had a patient global assessment >1, suggesting that these patients did not believe they were doing as well, or indeed were not doing as well as the objective criteria seemed to indicate, potentially attributable to their long disease duration.

Of the 255 patients who met Boolean remission criteria at week 36, 250 patients (98.0 %) were concordant, 5 (2.0 %) were negatively discordant, and none were positively discordant. However, of the 187 patients who had SJC and TJC ≤1, CRP ≤1 mg/dL, and patient global assessment >1, only 93 (49.7 %) were concordant; 2 (1.1 %) were negatively discordant, and 92 (49.2 %) were positively discordant. The patients who met Boolean remission criteria demonstrated greater improvement in clinical and patient-reported outcomes between baseline and week 36 than the patients with SJC and TJC ≤1, CRP ≤1 mg/dL, and patient global assessment >1. This was particularly true for the outcomes of patient general health and FACIT-fatigue (data not shown).

In comparison, 205/762 patients (26.9 %) achieved CDAI remission at week 36. Of those patients, 187 (91.2 %) were concordant, 2 (1.0 %) were negatively discordant, and 16 (7.8 %) were positively discordant. These results are similar to those for Boolean remission, with the exception that more patients in CDAI remission were positively discordant.

### Relationship between discordance status and remission

An additional analysis found that for patients with positive discordance, concordance, and negative discordance at baseline, 44/194 (22.7 %), 193/520 (37.1 %), and 18/48 (37.5 %), respectively, achieved Boolean remission at week 36, and 44/193 (22.8 %), 145/520 (27.9 %), and 16/48 (33.3 %), respectively, achieved CDAI remission. In comparison, for patients with positive discordance, concordance, and negative discordance at week 36, 0/189 (0 %), 250/551 (45.4 %), and 5/18 (27.8 %), respectively, achieved Boolean remission at week 36 and 16/189 (8.5 %), 187/555 (33.7 %), and 2/18 (11.1 %), respectively, achieved CDAI remission.

### Correlations and predictors of discordance

The baseline values of BPI, SJC, duration of morning stiffness, FACIT-fatigue, and patient general health significantly correlated with week-36 discordance, *p* < 0.0001 to *p* < 0.05 (Table 3), although the correlations were weak (*r* <0.25). At week 36, DAS28, duration of morning stiffness, HAQ-DI, FACIT-fatigue, CDAI, and SDAI correlated significantly but weakly with discordance, *p* < 0.0001 for all. BPI and patient general health demonstrated the strongest correlations, which were moderate, at week 36 (*r* = 0.48 and 0.58, respectively, *p* < 0.0001 for both).

**Table 3:** Correlation between week-36 discordance and measurements of disease at baseline and week 36, and change from baseline to week 36

	Baseline		Week 36		Change baseline to week 36	
	Number	Pearson’s *r*	Number	Pearson’s *r*	Number	Pearson’s *r*
DAS28	760	−0.0225	762	0.1832***	760	0.1942***
CRP	754	−0.0561	756	−0.0097	754	0.0523
ESR	763	−0.0630	763	0.0287	763	0.0924*
TJC	760	0.0058	762	0.0449	760	0.0269
SJC	760	−0.1197**	762	−0.0662	760	0.0678
Duration of morning joint stiffness	745	0.0813*	747	0.1456***	745	−0.0094
BPI	753	0.2268***	761	0.4784***	753	0.1854***
HAQ-DI	757	0.0648	762	0.3174***	757	0.2404***
FACIT-fatigue	753	−0.1437***	761	−0.3645***	753	−0.2054***
CDAI	760	0.0116	762	0.1986***	760	0.1602***
SDAI	751	−0.0068	755	0.1912***	751	0.1698***
Patient general health VAS	762	0.1826***	763	0.5784***	762	0.3542***
Duration of disease	761	−0.0402	na	na	na	na

Discordance is defined as patient global assessment – physician global assessment. *BPI* brief pain inventory, *CDAI* clinical disease activity index, *CRP* C-reactive protein, *DAS28* disease activity score based on 28 joint count, *ESR* erythrocyte sedimentation rate, *FACIT-fatigue* functional assessment of chronic illness therapy-fatigue, *HAQ-DI* health assessment questionnaire disability index, *na* not available, *SDAI* simplified disease activity index, *SJC* swollen joint count, *TJC* tender joint count, *VAS* visual analog scale. *p < 0.05, **p < 0.001, ***p < 0.0001

Baseline predictors of week-36 positive discordance were patient general health, BPI, and CRP. The odds ratios (95 % confidence interval) were similar for the full population (patient general health: 1.02 (1.00, 1.03), BPI: 1.22 (1.11, 1.35), CRP: 0.98 (0.97, 1.00)) and the subpopulation of patients who achieved SJC and TJC ≤1 and CRP ≤1 mg/dL at week 36 (patient general health: 1.03 (1.01, 1.04), BPI: 1.24 (1.07, 1.43), CRP: 0.97 (0.94, 1.00)).

### WPAI scores

At baseline, mean percent WPAI activity impairment was higher (greater impairment) for the patients with positive discordance (51.1 %) than for the patients with concordance (41.3 %) or negative discordance (42.8 %) (*p* < 0.0001 across means) (Table 4). This continued to week 36, with mean activity impairment of 35.4 %, 15.1 %, and 25.6 % for the positive discordance, concordant, and negative discordance groups, respectively, *p* < 0.0001. The greatest improvement between baseline and week 36 occurred in the patients who were concordant at week 36. For the subgroup of patients who were employed at baseline and 36 weeks and had WPAI data (n = 287), the mean percent impairment while working was higher for the patients with positive discordance at baseline and 36 weeks (50.0 % and 26.3 %, respectively) than for the patients with concordance (37.8 % and 10.7 %, respectively) or negative discordance (42.0 % and 12.0 %, respectively) (*p* ≤ 0.0026 across means) (Table 4). Similarly, the mean percent overall work impairment was higher for the patients with positive discordance at baseline and 36 weeks (54.6 % and 28.7 %, respectively) than for the patients with concordance (41.5 % and 12.2 %, respectively) or negative discordance (43.7 % and 12.0 %, respectively) (*p* ≤ 0.0019 across means). Mean percent work time missed did not differ significantly between the concordance categories; it was numerically highest in the positive discordance group at both baseline and week 36 (Table 4).

**Table 4:** Work productivity activity impairment questionnaire for rheumatoid arthritis (WPAI:RA) results at baseline, week 36, and change from baseline to week 36, according to week-36 concordance category

All patients		Positive discordance (n = 187)		Concordance (n = 549)		Negative discordance (n = 18)		
		Mean (SD)	Median (Q1, Q3)	Mean (SD)	Median (Q1, Q3)	Mean (SD)	Median (Q1, Q3)	*P* value^a^
Activity impairment (n = 754)	Baseline, %	51.1 (20.7)	50.0 (40.0, 70.0)	41.3 (19.2)	40.0 (30.0, 50.0)	42.8 (19.9)	45.0 (30.0, 60.0)	<0.0001
	Week 36, %	35.4 (20.8)	30.0 (20.0, 50.0)	15.1 (16.6)	10.0 (0, 20.0)	25.6 (21.2)	20.0 (10.0, 50.0)	<0.0001
	Δ Baseline to week 36	−15.7 (24.5)	−10.0 (–30.0, 0)	−26.2 (21.7)	−30.0 (–0.0, –10.0)	−17.2 (25.9)	−10.0 (–30.0, 0)	<0.0001
Employed patients		Positive discordance (n = 76)		Concordance (n = 206)		Negative discordance (n = 5)		
		Mean (SD)	Median (Q1, Q3)	Mean (SD)	Median (Q1, Q3)	Mean (SD)	Median (Q1, Q3)	*P* value^a^
Impairment while working (n = 287)	Baseline, %	50.0 (27.4)	40.0 (30.0, 70.0)	37.8 (25.5)	30.0 (20.0, 50.0)	42.0 (27.7)	50.0 (20.0, 50.0)	0.0026
	Week 36, %	26.3 (18.7)	20.0 (20.0, 40.0)	10.7 (13.8)	10.0 (0, 20.0)	12.0 (13.0)	10.0 (0, 20.0)	<0.0001
	Δ Baseline to week 36	−23.7 (29.9)	−20.0 (–0.0, 0)	−27.1 (26.4)	−20.0 (–40.0, –10.0)	−30.0 (23.5)	−20.0 (–50.0, –10.0)	<0.0001
Overall work impairment (n = 287)	Baseline, %	54.6 (27.1)	50.0 (33.2, 76.1)	41.5 (27.2)	40.0 (20.0, 60.0)	43.7 (28.6)	50.0 (20.0, 58.3)	0.0019
	Week 36, %	28.7 (20.6)	20.0 (20.0, 40.0)	12.2 (15.9)	10.0 (0, 20.0)	12.0 (13.0)	10.0 (0, 20.0)	<0.0001
	Δ Baseline to week 36	−25.8 (30.9)	−20.1 (–45.0, –8.6)	−29.3 (27.4)	−22.0 (–46.7, –10.0)	−31.7 (25.4)	−20.0 (–58.3, –10.0)	<0.0001
Employed patients		Positive discordance (n = 74)		Concordance (n = 200)		Negative discordance (n = 5)		
		Mean (SD)	Median (Q1, Q3)	Mean (SD)	Median (Q1, Q3)	Mean (SD)	Median (Q1, Q3)	*p* value^a^
Work time missed (n = 279)	Baseline, %	16.9 (30.2)	0 (0, 17.2)	10.7 (24.3)	0 (0, 6.3)	3.3 (7.5)	0 (0, 0)	0.1565
	Week 36, %	5.9 (15.9)	0 (0, 1.8)	2.4 (11.0)	0 (0, 0)	0 (0)	0 (0, 0)	0.2608
	Δ Baseline to week 36	−11.0 (30.9)	0 (–10.5, 0)	−8.2 (22.9)	0 (–2.5, 0)	−3.3 (7.5)	0 (0, 0)	0.2608

Activity impairment includes all patients who had both baseline and week 36 data for this parameter. The remaining WPAI parameters include only patients who were employed and had WPAI data at both baseline and week 36. A total of 305 patients were employed at both baseline and week 36; individual WPAI parameters differ due to missing baseline or week 36 values. Positive discordance: patient global assessment – physician global assessment ≥2. Negative discordance: patient global assessment – physician global assessment ≤ –2. Concordance: patient global assessment – physician global assessment = 0 or 1. *Δ* change; *Q1* quartile 1, *Q3* quartile 3. ^a^ *P* value for baseline is for one-way analysis of variance, and for week 36 it is for an analysis of covariance model adjusted for baseline of the parameter

## Discussion

We evaluated the rates of concordance and discordance of global disease activity assessments at baseline and after 36 weeks of open-label, single-arm treatment with etanercept and methotrexate in patients with moderate RA who participated in the PRESERVE trial. At baseline, 31.8 % of patient assessments of disease activity were discordant with the assessments of their physicians. This represents a disconnect between the patient and physician in verbal and non-verbal cues about how RA is affecting the patient. In most cases, discordance reflected higher ratings of disease activity by patients.

When comparing concordance in this study to others that used a similar method of measurement, the rate of concordance was comparable to several [1, 3, 4] and higher than other studies [2, 5]. As with other studies, we found that pain was an important contributor to the patient global assessment and discordance [2–5, 9, 11–13, 15]. We also noted that patients with relatively high levels of inflammatory activity, as assessed by an objective measure such as CRP, had a higher level of concordance than patients with lower levels of inflammation, similar to several other studies [1, 4, 5, 9, 12]. In our analysis, baseline CRP was highest in patients with concordance, with a mean of 13.4 mg/L, compared with 9.9 mg/L for positive discordance and 8.1 mg/L for negative discordance. This suggests that patients with high levels of inflammation tend to focus on indicators of potential joint damage, similar to clinicians.

At week 36, the rate of discordance was 27.1 % overall. Among the subgroup of 442 patients who achieved SJC and TJC ≤1 and CRP ≤1 mg/dL, the discordance rate was lower but still substantial at 22.4 %. This result provides additional evidence that even when the disease is controlled, pain may still be present, resulting in a considerable difference between patient and physician viewpoints [15]. Not surprisingly, for the 255 patients in this subgroup who also reported a patient global assessment ≤1 (thereby achieving a Boolean remission), the rate of discordance was 2 %. This is entirely negative discordance, and is comparable to the negative discordance of 2.4 % for the overall population. The overall decrease in discordance from 31.8 % at baseline to 27.1 % at week 36 occurred in the context of improved disease control. This may be a result that is independent of the gap in communication, or it may be due to the physician becoming more adept at interpreting patient cues, or patients becoming more expressive of how they feel.

This analysis of the PRESERVE trial provides a novel perspective in several ways. First, it focuses on patients with moderate RA (DAS28 >3.2 and ≤5.1) who received therapy with etanercept and methotrexate. Many previously reported studies did not specify the extent of disease activity, nor did they examine discordance following treatment with a tumor necrosis factor inhibitor. As the majority of patients seen in clinical practice have low or moderate RA activity [36, 37], the data presented in this report are relevant to most patients.

We also assessed the relationship between discordance, activity impairment, and work productivity. Previous studies have found that moderate RA (and even mild RA) is associated with work impairment [23, 24, 27]. Additionally, one study of patients with RA and other inflammatory and noninflammatory rheumatic diseases found a significant association between discordance and not working [2]. We examined this association more closely by using the WPAI:RA to measure four components of work productivity: activity impairment, absenteeism, presenteeism, and overall work impairment. All three components of the WPAI:RA related to employment were highest (i.e., worst) in patients with positive discordance; for two of these components the difference was statistically significant. These results suggest that discrepancies between patient and physician assessments of disease not only are associated with poorer clinical outcomes, but may also extend to other domains of a patient’s life such as work productivity, which could potentially have an economic impact on patients and society.

A strength of this study is that it evaluated data from a clinical trial population, thereby ensuring that all patients were managed in a consistent manner and data were collected at fixed intervals. This includes collection of work productivity data using a validated instrument, the WPAI:RA. Additionally, investigators completed the physician global assessment prior to having access to CRP values for that visit. This removed the potential for the CRP results to bias the physician assessment. In the clinic, patients sometimes bring their CRP or ESR results with them. Therefore, the current study may more accurately reflect the similarities and differences between patient global assessments and physician global assessments than in the clinic [9, 38].

This study has several limitations. Importantly, we were limited to the assessments that were used in the clinical trial. Although of interest, we were not able to evaluate the effect of depression or cognitive impairment on discordance because no validated measurement tool was used in the trial. In addition, as this was a clinical trial, the results may not be generalizable to all patients with RA. This study excluded patients with mild or severe disease activity and those with certain comorbid conditions. As a clinical trial, physicians could not modify medication regimens at will; instead it was necessary to follow the study protocol. Also, patients who left the study for any reason were not followed, and so we were unable to determine whether their concordance status changed over time.

## Conclusions

The results of our analysis demonstrated that in patients with moderately active RA, the rate of concordance of patient and physician global assessments increased after 36 weeks of treatment with etanercept and methotrexate. Discordance significantly correlated with several clinical endpoints and was associated with decreased work productivity. Additional research into verbal and nonverbal patient/physician communication is needed to fully understand the discrepancies.

## Abbreviations

BPI: brief pain inventory
CDAI: clinical disease activity index
CRP: C-reactive protein
DAS28: disease activity score based on 28-joint count
DMARD: disease-modifying anti-rheumatic drug
ESR: erythrocyte sedimentation rate
FACIT-fatigue: functional assessment of chronic illness therapy-fatigue
HAQ-DI: health assessment questionnaire disability index
RA: rheumatoid arthritis
SDAI: simplified disease activity index
SJC: swollen joint count
TJC: tender joint count
VAS: visual analog scale
WPAI: work productivity activity impairment questionnaire
